# Genome-wide identification, evolution and function analysis of *UGTs* superfamily in cotton

**DOI:** 10.3389/fmolb.2022.965403

**Published:** 2022-09-13

**Authors:** Liangqing Sun, Lanjie Zhao, Hui Huang, Yuexin Zhang, Junjuan Wang, Xuke Lu, Shuai Wang, Delong Wang, Xiugui Chen, Chao Chen, Lixue Guo, Nan Xu, Hong Zhang, Jing Wang, Cun Rui, Mingge Han, Yapeng Fan, Taili Nie, Wuwei Ye

**Affiliations:** ^1^ Institute of Cotton Research of Chinese Academy of Agricultural Sciences/Zhengzhou Research Base, State Key Laboratory of Cotton Biology, School of Agricultural Sciences, Zhengzhou University, Anyang, China; ^2^ Cotton Research Institute of Jiangxi Province, Jiujiang, China

**Keywords:** gene family, UDP-glycosyltransferase, evolution, gene function, cotton

## Abstract

Glycosyltransferases mainly catalyse the glycosylation reaction in living organisms and widely exists in plants. *UGTs* have been identified from *G. raimondii*, *G. arboreum* and *G. hirsutum*. However, Genome-wide systematic analysis of *UGTs* superfamily have not been studied in *G. barbadense*. 752 *UGTs* were identified from four cotton species and grouped into 18 clades, of which R was newly discovered clades. Most *UGTs* were clustered at both ends of the chromosome and showed a heterogeneous distribution. UGT proteins were widely distributed in cells, with the highest distribution in chloroplasts. *UGTs* of the same clade shared similar intron/exon structural features. During evolution, the gene family has undergone strong selection for purification. *UGTs* were significantly enriched in “transcriptional activity (GO:0016758)” and “metabolic processes (GO:0008152)”. Genes from the same clade differed in function under various abiotic stresses. The analysis of cis-acting element and qRT–PCR may indicate that *GHUGT*s play important roles in plant growth, development and abiotic stress. We further found that *GHUGT74-2* plays an important role under submergence. The study broadens the understanding of *UGTs* in terms of gene characteristics, evolutionary processes, and gene function in cotton and provides a new way to systematically and globally understand the structure–function relationship of multigene families in the evolutionary process.

## Introduction

Glycosyltransferases (GTs) mainly catalyse the glycosylation reaction in living organisms, and transfers the sugar group from the activated donor molecule to the acceptor molecule, thereby forming a variety of glycoside compounds (Vogt and Jones, 2000). Glycosyltransferases are a highly differentiated family of superenzymes. According to the similarity of glycosyltransferase sequences, the specificity of the catalytic substrate and the stereochemical structure of the catalytic product, the online website CAZy (http://www.cazy.org) has divided GTs into 114 families (GT1∼GT114) by the end of 2021 (Campbell et al., 1997; Coutinho et al., 2003). Uridine diphosphate-glucose is the most common plant glycosyl donor, and plant glycosyltransferases are also known as UGTs (Hughes and Hughes, 1994; Ross et al., 2001). The glycosyl acceptor substrates of glycosyl-transferases in plant are diverse, including not only monosaccharides, oligosaccharides, and polysaccharides but also various noncarbohydrates, such as proteins, antibiotics, and lipids (Keegstra and Raikhel, 2001; Ross et al., 2001; Bowles et al., 2005; Wilson and Tian, 2019). Glycosylation directly affects biological activity and stability of compounds (Li et al., 2001; Lepak et al., 2015).

*UGT* family genes have a conserved sequence of 44 amino acids in the C-terminal region, known as the plant secondary product glycosyltransferase (PSPG) motif (Ross et al., 2001; Yonekura-Sakakibara and Hanada, 2011). Genome-wide analysis has identified numerous UGT family members in multiple species, including 107 in *Arabidopsis* (Ross et al., 2001), 147 in *Zea mays* (Li et al., 2014), 96 in *Cicer arietinum* (Sharma et al., 2014), 137 in *Linum usitatissimum* (Barvkar et al., 2012), 130 in *Prunus mume* (Zhang et al., 2018), 179 in *Triticum aestivum* (He et al., 2018), and 121 in *Manihot esculenta* (Wu et al., 2021). The Arabidopsis *UGT* gene family is divided into 14 distinct clades (A-N) (Li et al., 2001; Ross et al., 2001). The O clade and P clade were found in *Triticum aestivum*, *Zea mays*, etc., (Li et al., 2014; He et al., 2018). The O clade is primarily responsible for the glycosylation of plant hormones (Caputi et al., 2012). The group Q of UGTs was lost in Poales and Brassicales (Wilson and Tian, 2019). With the mining of new *UGTs*, the evolutionary classification of *UGT* gene families is constantly being improved. The gene and function of the *UGTs* superfamily in 65 species have been studied by Alexander E. (Wilson and Tian, 2019).

UGTs play important roles in response to plant growth and development, secondary metabolism and stress (Jones and Vogt, 2001; Bowles et al., 2005). The first glycosyltransferase gene discovered in plants was flavonoid glucosyltransferase (*UFGT*) (Dooner and Nelson, 1977). Plant *UGTs* can modify plant endogenous hormones by glycosylation, affecting the content and activity of plant endogenous hormones and thereby regulating plant growth and development. Glycosyltransferases related to auxin (*UGT75D1*, *UGT84B1*, *UGT74D1*) (Zhang et al., 2016; Aoi et al., 2020; Mateo-Bonmatí et al., 2021), abscisic acid (*UGT71B6*) (Priest et al., 2006), and brassinolide (*UGT73C5*) (Poppenberger et al., 2005) have been identified in plants. Some glycosyltransferases related to stress were identified by gene silencing or overexpression of *UGT* in plants, such as those related to salt stress (*UGT85A5*) (Sun et al., 2013), cold stress resistance (*UGT80B1*) (Mishra et al., 2015) and drought stress (*UGT87A2*) (Li et al., 2017).

Cotton is an important model crop for studying the evolution of plant polyploidy and supergene family. A total of 142, 146 and 196 *UGTs* have been identified from *G. raimondii*, *G. arboreum* and *G. hirsutum*, respectively (Huang et al., 2015). However, there have been no studies on the genome-wide analysis of *UGTs* from *G. barbadense* or reports on the structure–evolutionary function of the cotton *UGT* gene family. Based on newly released cotton genome data, we identified members of the *UGT* gene family from four cotton species. Bioinformatic methods were used to comprehensively analyse the physicochemical properties, gene structure, chromosome distribution, phylogeny, gene duplication, collinearity/syntaxy and expression profiles of *UGTs* under different tissues and stresses in cotton. In conclusion, the study of the *UGTs* superfamily provides new insights into the systematic and global understanding of the structure–function relationship of multigene families during evolution.

## Results

### Identification of UGT family members

**FIGURE 1:**
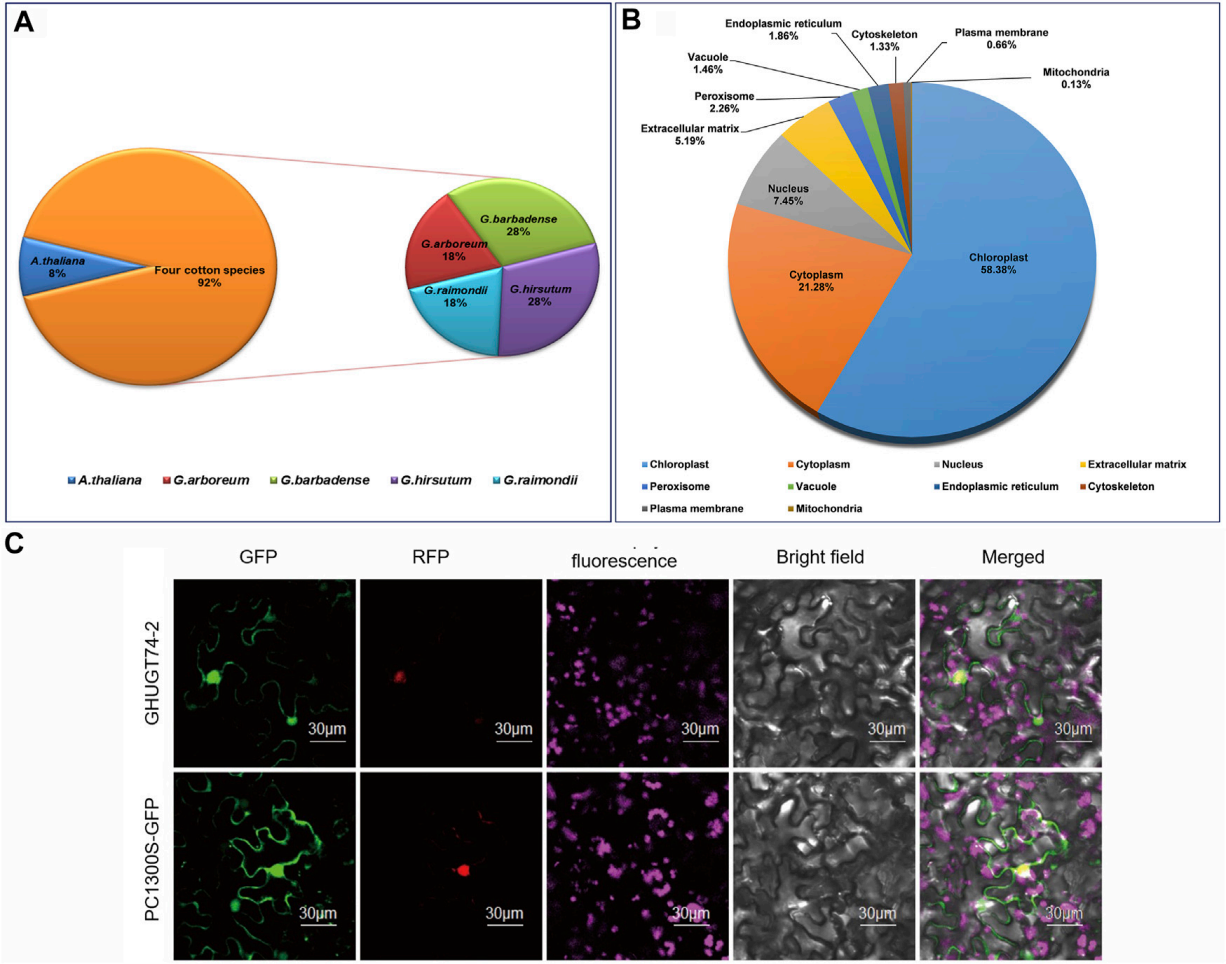
**(A)** Distribution of 818 *UGTs* from five plant species. **(B)** Subcellular localization of 752 UGT proteins from four cotton species. **(C)** Subcellular localization of GHUGT74-2 proteins carried out in tobacco leaves.

The hidden Markov model (HMM) file of PF00201.20 was used as a query tool to search for *UGTs* in *G. hirsutum*, *G. arboreum*, *G. raimondii*, *G. barbadense* and *A. thaliana* with an e value of 1e-5. We identified 818 *UGTs* from five different plant species, of which 752 *UGTs* were evaluated from four cotton species. 226, 228, 146, and 152 *UGTs* were identified from *G. hirsutum*, *G. barbadense*, *G. arboreum* and *G. raimondii*, respectively (Figure 1). We renamed the *UGTs* of five plant species (Supplementary Table S1). We further analysed the *UGT* gene of four cotton species, including gene location, gene length, transcript length, length and GC content of CDS, mean exons/intron length, protein length, molecular weight (MW), charge, and isoelectric points (pI) (Supplementary Table S2). Taking the most widely used polyploid model plant, *G. hirsutum*, as an example, we analysed its biophysical properties. All 226 *UGTs* encoded proteins varying from 341 (*GHUGT71-12*) to 963 (*GHUGT71-10*) amino acids. The pI varied from 4.804 (*GHUGT74-10*) to 8.296 (*GHUGT89-11*) with a mean of 6.283, and the MWs ranged from 38.149 kDa (*GHUGT83-10*) to 108.765 kDa (*GHUGT71-10*). A total of 78.89% of the 226 GHUGT proteins were hydrophobic, while 26.11% were hydrophilic. Other information on gene location, gene length, transcript length, length and GC content of CDS, exon number, mean exons/intron length and charge of every *GHUGT* is given in Supplementary Table S2. The prediction of subcellular localization indicates that there are 439 UGT proteins located in the chloroplast, 160 in the cytoplasm, 56 in the nucleus, 39 in the extracellular matrix, 17 in the peroxisome, 14 in the endoplasmic reticulum, 11 in the vacuole, 10 in the cytoskeleton, five in the plasma membrane and one in the mitochondria (Figure 1B). We took GHUGT74-2 proteins as an example and found that GHUGT74-2 proteins were located in the nucleus (Figure 1C).

### Phylogenetic analysis of *UGTs*

**FIGURE 2:**
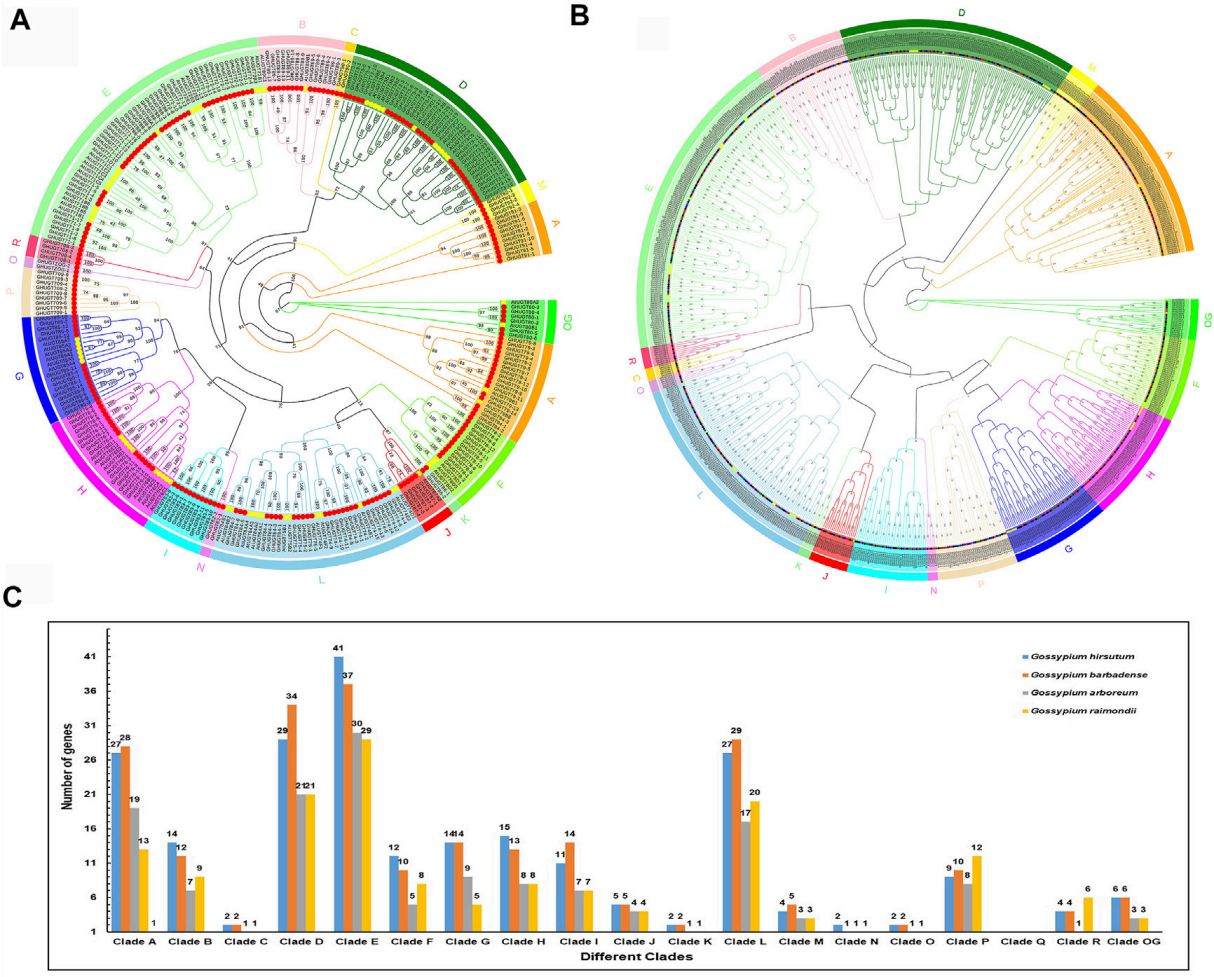
*UGT* gene family phylogenetic tree in five plant species. **(A)** Phylogenetic tree of 292 *UGTs* from *A. thaliana* and *G. hirsutum*. **(B)** Phylogenetic relationship of 752 identified *UGTs* from four cotton species. **(C)** Distribution of *UGT*s into their different clades from five plant species.

To study the evolutionary history of *UGTs* in cotton, we constructed an unrooted phylogenetic tree by Maximum Likelihood (ML) method (Figure 2). We constructed a GHUGTs phylogenetic tree of *G. hirsutum* based on the taxonomic relationship of the *A. thaliana* UGT protein family (Wilson and Tian, 2019). A total of 226 *GHUGTs* were classified into 18 clades, and Clade E had the highest proportion (41 *GHUGTs*). The distribution of other *UGT*s among different clades was as follows: D (29), A (27), L (27), H (15), B (14), G (14), F (12), I (11), P (9), OG (6), J (5), M (4), R (4), C (2), K (2), N (2), O (2) and Q (0) (Figure 2C). Similarly, we built another unrooted tree of 752 *UGT* proteins from *Gossypium* with a similar method (Figure 2B). A total of 752 *UGTs* were classified into 18 clades according to the criterion proposed by Alexander E. (Wilson and Tian, 2019), which were unevenly distributed (Figure 2B). The *UGTs* of these four species were present in almost every clade. Among them, Clade E contained the most *UGT* members, and Clade N had the fewest members. The number of *UGT* members in Clades E and N was distributed as 137 and 5, accounting for 18.21% and 0.66% of the number of *UGTs*, respectively. Clade Q was lost. The distribution of other *UGT* members among different clades was as follows: D (105), L (93), A (87), H (44), G (42), B (42), P (39), I (39), F (35), OG (18), J (18), M (15), R (15), O (6), K (6), and C (6) (Figure 2C, Supplementary Table S5). Clade E contained the most members among the five plants, suggesting that it may be an ancient clade. Interestingly, *UGTs* in the four species had corresponding homologous genes in almost every clade, indicating that *UGTs* of these species are closely related to each other. The ratio of *UGTs* in allotetraploid *G. hirsutum* and *G. barbadense* was close to 1:1, and the ratio of *UGTs* in diploid *G. raimondii* and *G. arboreum* was also close to 1:1. The ratio of *UGTs* in allotetraploid cotton and diploid cotton was less than 2:1, which may be the result of evolutionary selection in the process of hybridizing two diploid cotton plants to form allotetraploid cotton.

### Chromosomal localization of *UGTs*

**FIGURE 3:**
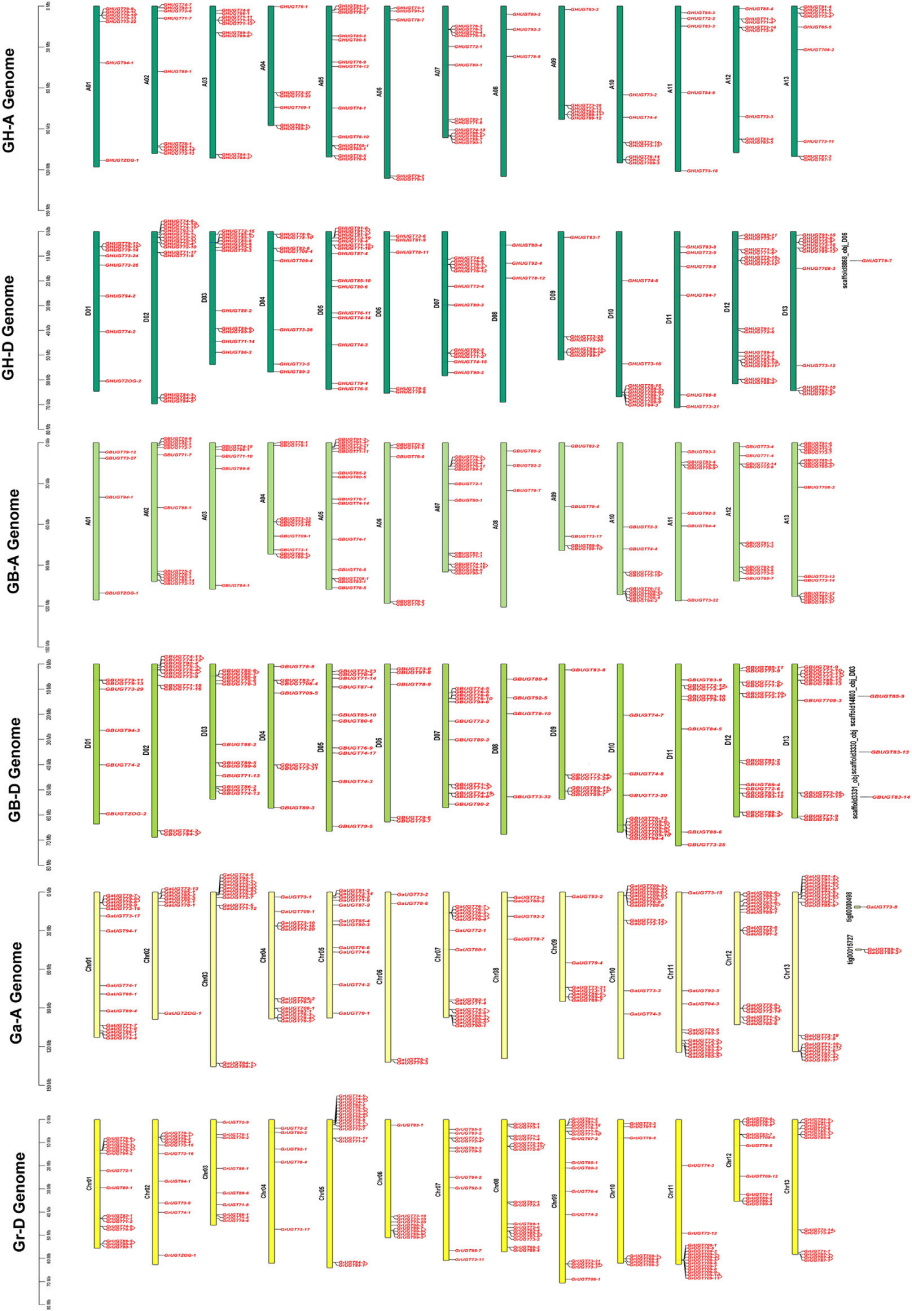
Location of *UGTs* in four cotton species. Gene pairs have been annotated on the chromosome with gene ID. The vertical bars represent the location of *UGTs* and the length of chromosomes.

To understand the physical location of *UGTs* on chromosomes, chromosome maps of 752 *UGTs* were constructed in four cotton species (Figure 3). A total of 745 of the 752 *UGTs* were located to specific chromosomes, the other seven *UGTs* were assigned to unmapped scaffolds, indicating a higher evolutionary maturity of the *UGT* family (Figure 3, Supplementary Table S6). Most *UGTs* were distributed on both ends of chromosomes. In addition, the number of genes in the heterologous tetraploid cotton (*G. hirsutum* and *G. barbadense*) At/Dt subgenome was less than the number of genes in the diploid cotton A/D genome (Supplementary Figure S1, Supplementary Table S6), indicating that the *UGT* family may have been lost during evolution. Two genes were clustered together to form a gene pair, forming a total of 146 gene pairs, including 40 in *G. hirsutum*, 31 in *G. barbadense*, 41 in *G. arboreum*, and 34 in *G. raimondii* (Figure 3). This indicated that gene duplication occurred during the evolution of the *UGT* family.

Among 226 *UGTs* identified in *G. hirsutum*, 100 members were located on 13 chromosomes on the At subgenome (GHAt), 125 *UGTs* were distributed on 13 chromosomes on the Dt subgenome (GHDt), and one *UGT* was on a scaffold (Figure 3). For subgenome GHAt, GHAt-05 and GHAt-07 had the most *UGT* members (13), while GHAt-06 and GHAt-11 had the least number of genes (5). For the subgenome GHDt, GHDt-12 had the largest number of *UGT* members (16), while GHDt-08 had the smallest number of *UGT* members (3) (Supplementary Figure S1A, Supplementary Table S6). Similarly, 225 out of 228 *UGTs* from *G. barbadense* were mapped on their 26 chromosomes, and the remaining three *UGTs* were on scaffolds. The subgenome (GBAt) contained 109 genes, and the Dt subgenome (GBDt) contained 116 *UGTs* (Figure 3). For the GBAt subgenome, GBAt-05 and GBAt-13 had the most *UGT* members (14), while GBAt-08 had the least number of genes (3). For the GBDt subgenome, GBDt-12 had the largest number of *UGT* members (14), while GBDt-08 had the smallest number of *UGT* members (3) (B, Supplementary Table S6). In *G. arboreum*, 143 genes were mapped to chromosomes, and three *UGTs* were not annotated to chromosomes (Figure 3). Chr13 (A13), Chr12 (A12) and Chr07 (A07) had more *UGTs* on chromosomes 18, 16, and 15, respectively. Chr06 (A06) and Chr08 (A08) had the least number of eight chromosomes (Supplementary Figure S1C, Supplementary Table S6). For *G. raimondii*, all 152 *UGTs* were distributed on 13 chromosomes (Figure 3). Chr08 (D08), Chr01 (D01) and Chr05 (D05) chromosomes had relatively more *UGTs*, 17, 16 and 16, respectively (Supplementary Figure S1D, Supplementary Table S6). In conclusion, the *UGTs* from the four cotton species were heterogeneously distributed on their chromosomes, with more genes located on the D genome/subgenome than on the A genome/subgenome.

### Sequence logos analysis of UGT proteins

**FIGURE 4:**
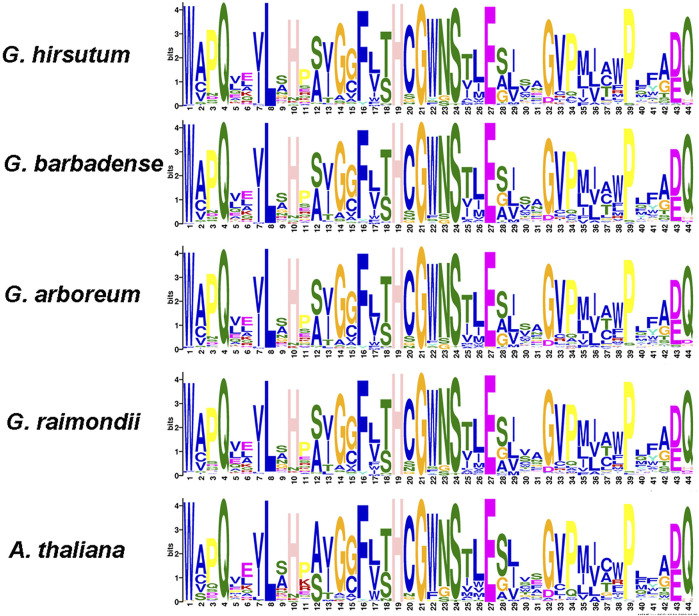
Consensus sequence logos of the PSPG-box in five plant species.

To study the evolutionary conservation of *UGT* genes in cotton, we constructed conserved amino acid sequence logos (Figure 4). We found a highly conserved sequence PSPG box consisting of 44 amino acid residues at the C-terminus of UGT sequences from five plant species (Mackenzie et al., 1997; Mackenzie et al., 2005) (Figure 4). Compared with consensus sequences, sequence logos describe sequence similarity more abundantly and more precisely and quickly reveal important features of sequence alignments (Crooks et al., 2004). Our results may indicate that the domain sequence of UGT is highly conserved in the five plant species.

### Conserved motif and exon–intron structure analysis in *G. hirsutum*

**FIGURE 5:**
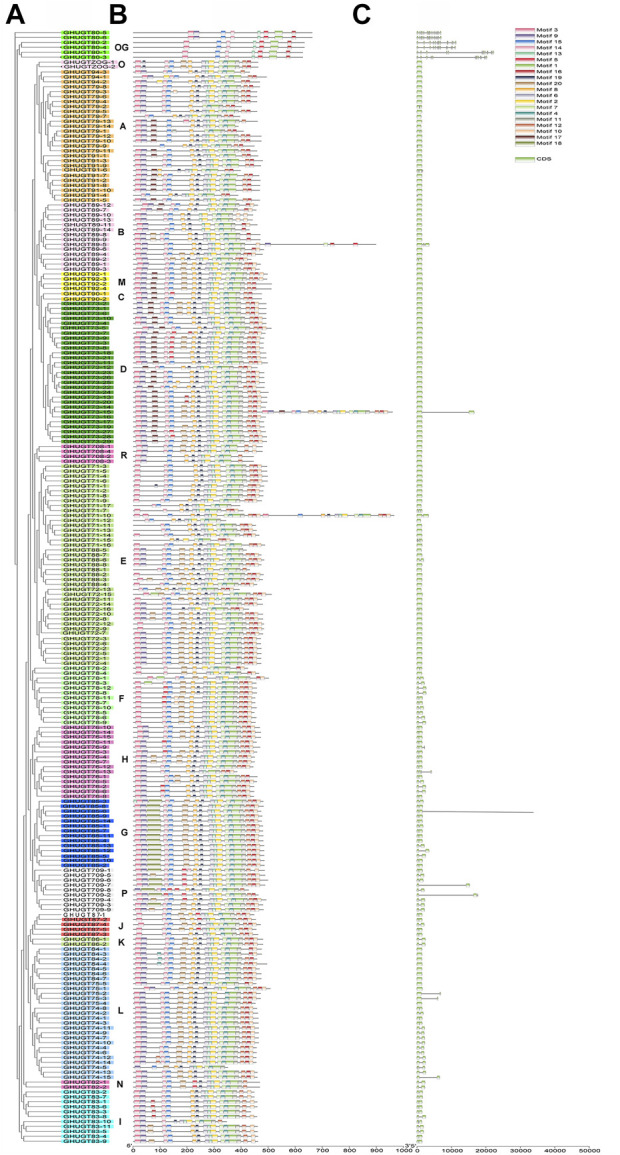
Phylogenetic tree, motifs and exon/intron structures of *GHUGTs* in *G. hirsutum*. **(A)** Phylogenetic tree of *GHUGTs*. **(B)** Conserved motifs of GHUGT proteins. **(C)** Exon/intron structures of *GHUGTs*.

Genes are composed of coding regions and noncoding regions. The structure and arrangement of introns and exons can be used to analyse the evolutionary relationship between members of different gene families. Previous studies have shown that the gene exon–intron structural features are related to their biological functions (Malik et al., 2020). In the *GHUGT* gene family, the gene sequence of the longest gene (*GHUGT85-6*) is approximately 93336 bp, while the shortest gene (*GHUGT71-12*) is only 1,026 bp. To further explore the possible structural evolutionary history of the *GHUGT* family, the phylogenetic and gene structure map of *G. hirsutum* were constructed (Figures 5A,C). Our results demonstrated that the distribution number of exon regions in *GHUGTs* varied from 1 to 15, while *GHUGTs* of the same clade had similar intron/exon structural features in terms of exon number and length (Figures 5A,C). Among the 226 *GHUGTs*, approximately 55.54% (121) had no introns, approximately 39.94% (88) had only one intron, and only 7.52% (17) had two or more introns (Figure 5C). The characteristics of fewer introns in *GHUGT* gene family members may indicate that this gene family was highly conserved. Overall, we found that *GHUGTs* had a strong evolutionary relationship between gene features and phylogeny, and displayed a conserved pattern of gene structure.

To further demonstrate the evolutionary relationships of *GHUGT* gene family members, we constructed a phylogenetic tree (Figure 5A) and motif distribution (Figure 5B) of all 226 GHUGT proteins. Our results showed that each GHUGT protein had various conserved motifs ranging from 7 to 35. GHUGT proteins with the same motif distribution pattern distributed in the same clade and clustered next to each other. Different clades had unique distribution patterns of conserved motifs. For example, Clade OG all had Motif 3, 15, 14, 13, 16, 1, and 5. Motif one was contained in all 18 evolutionary clades. The C-terminal protein sequence of GHUGT was more conserved than the N-terminal sequence. Overall, we found that the *GHUGT* gene family also had a strong evolutionary conserved pattern between gene structure and protein motif distribution.

### Duplication and collinearity relationship of *UGTs*

**FIGURE 6:**
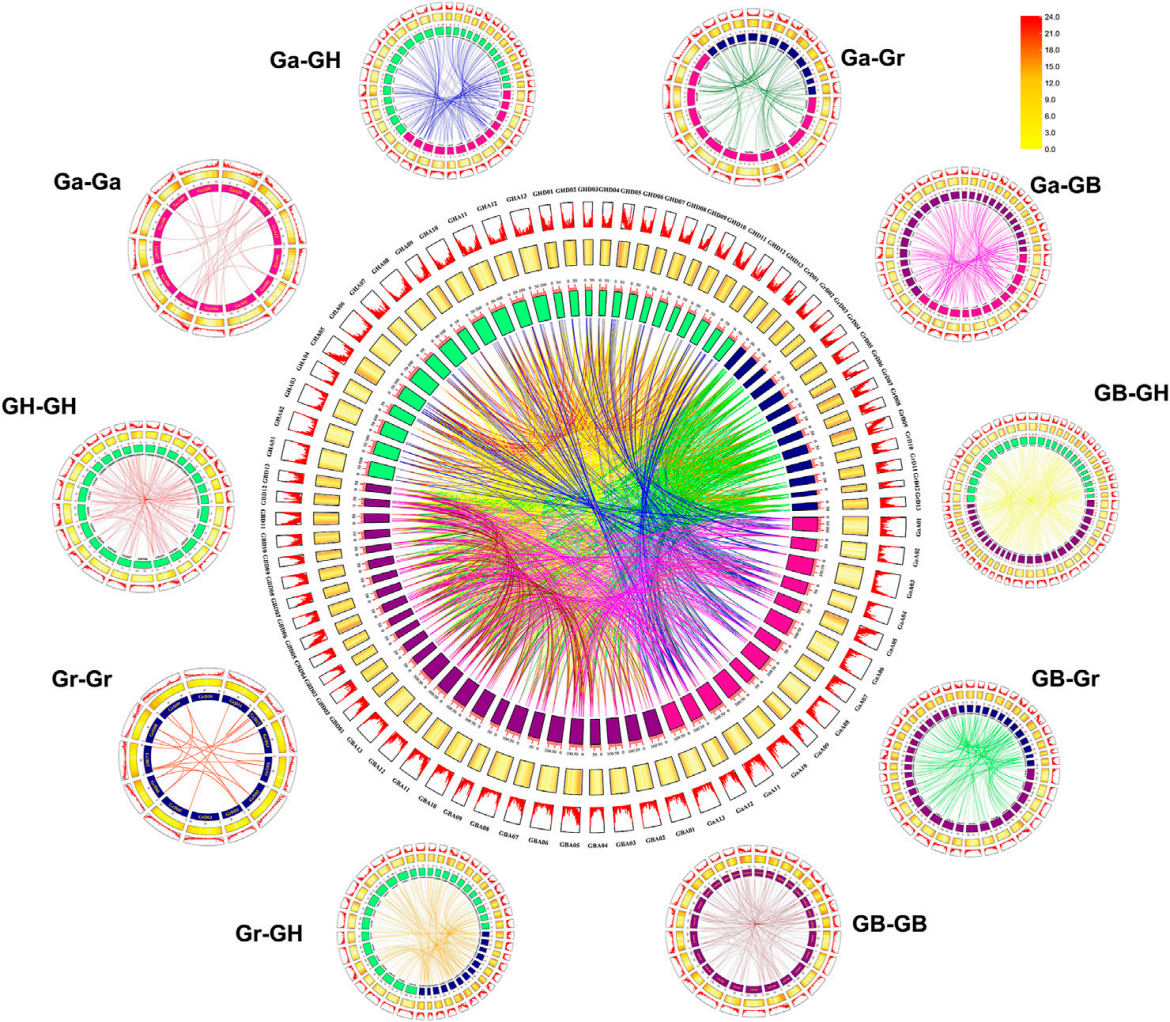
Syntenic relationship of *UGT* duplicated gene pairs in cotton. Chromosomal lines represented by different colours indicate various inter genome and intra genomic syntenic regions. The line map and heatmap of the outer rings represent the density of genes on chromosomes. *G. hirsutum* At subgenome “GHA”, *G. hirsutum* Dt subgenome “GHD”, *G. barbadense* At subgenome “GBA”, *G. barbadense* Dt subgenome “GBD”, *G. arboreum* A-genome “GaA”, and *G. raimondii* D-subgenome “GrD”.

**FIGURE 7:**
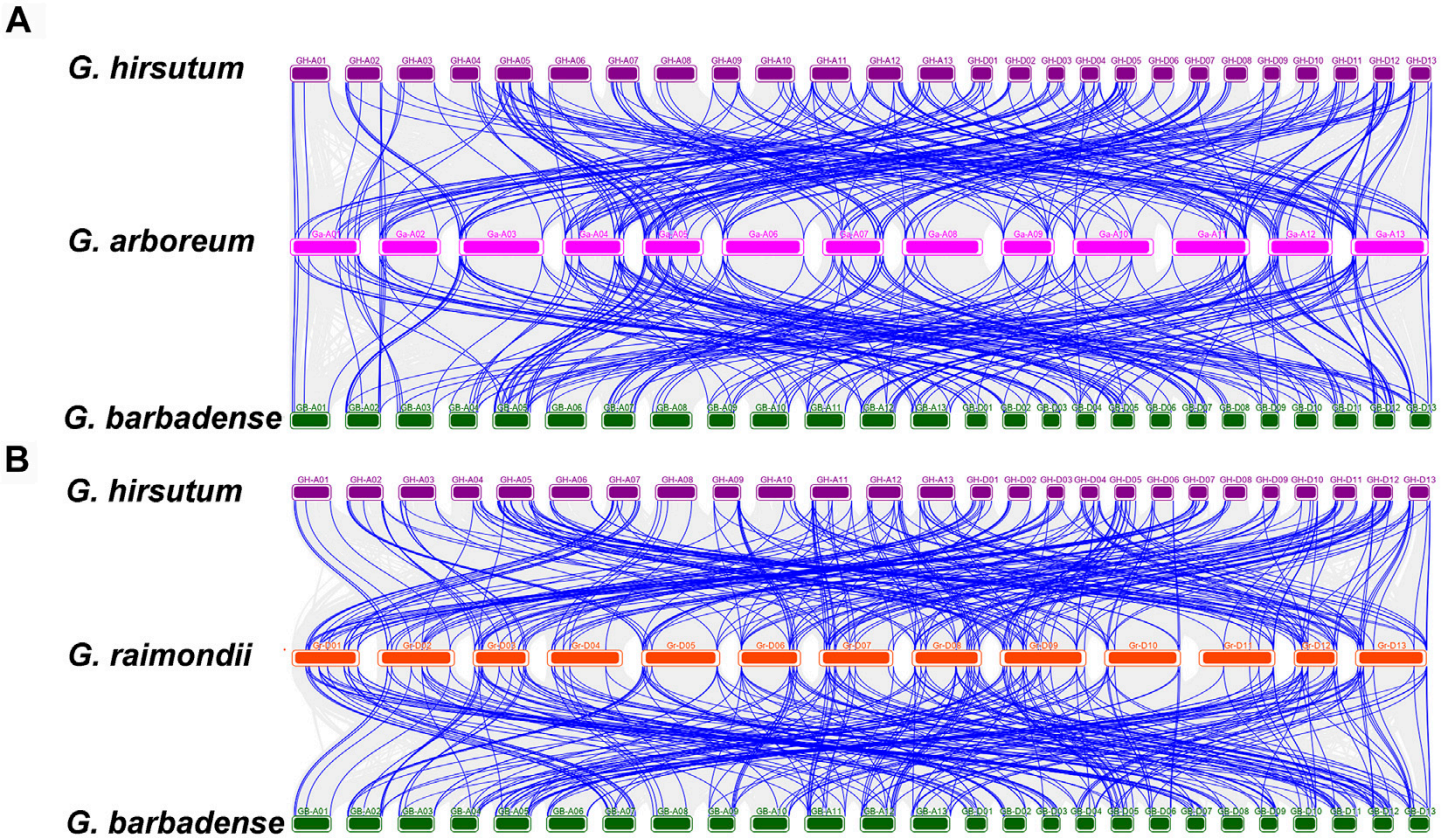
Multiple collinearity analysis of *GHUGTs* and *GBUGTs* compared with their ancestor species *G. arboreum* and *G. raimondii*. **(A)** Collinearity analysis of *GHUGTs* and *GBUGTs* compared with *G. arboreum*. **(B)** Collinearity analysis of *GHUGTs* and *GBUGTs* compared with *G. raimondii*. Dense grey lines in the background showed collinear blocks, while green lines revealed syntenic *UGT* gene pairs.

**FIGURE 8:**
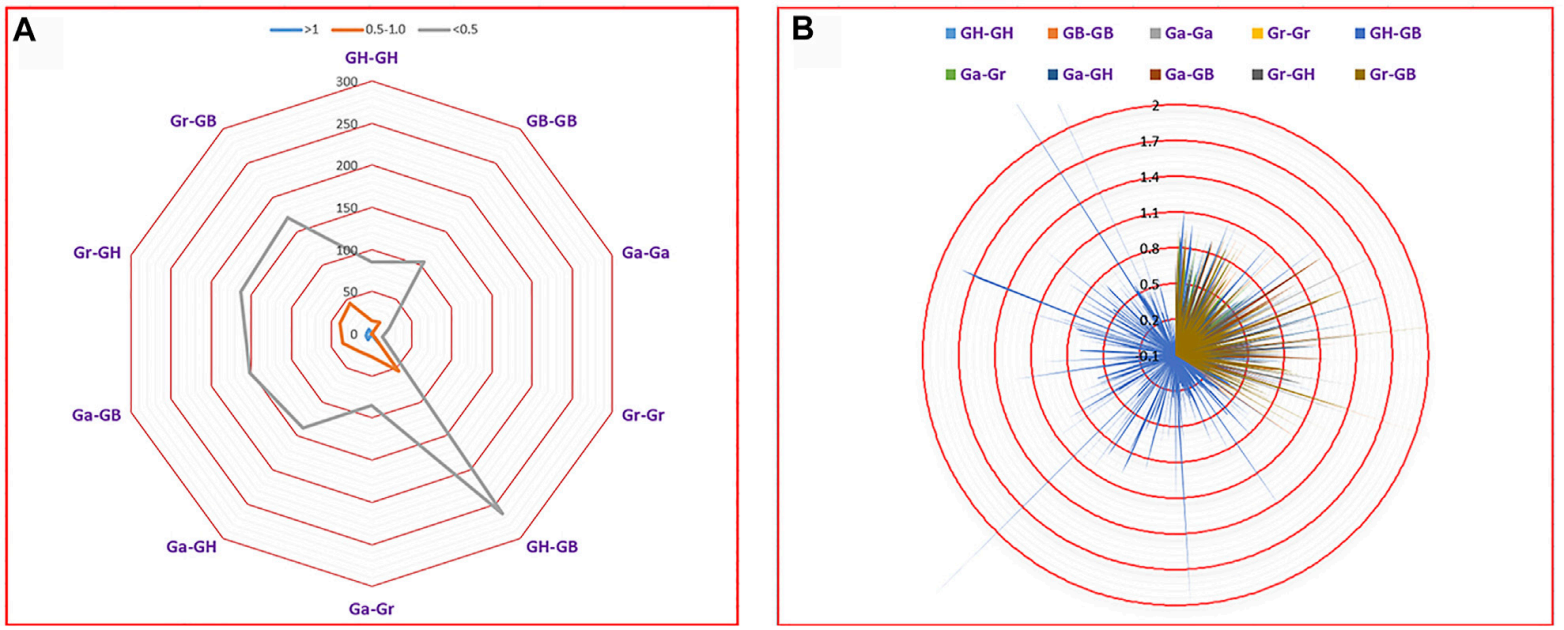
Selection pressure analysis based on Ka/Ks. **(A)** Number of duplicate gene pairs in different genomes of four cotton species. **(B)** Ka/Ks divergence values for GH-GH, GB-GB, Gr-Gr, Ga–Ga, GH-GB, Ga-Gr, Ga-GH, Ga-GB, Gr-GH and Gr-GB are displayed in a circular chart.

To understand the evolutionary relationship of *GHUGTs* from the diploid ancestral *G. arboreum* (A genome) and *G. raimondii* (D genome) and allotetraploid *G. hirsutum* and *G. barbadense* (AD genome), we constructed syntenic and collinear relationship maps of gene duplication pairs from four cotton species (Figures 7, 8). In this study, we obtained a total of 2,203 duplicated gene pairs, including 276 segmental duplications and 146 tandem duplications, in 752 *UGTs* from four cotton species (Figure 6, Supplementary Table S10). The remaining 1781 orthologous gene pairs underwent whole genome duplication (WGD), resulting in a large-scale expansion of the *UGT* gene family in cotton. Similar to previous findings, we also found that GHAt/GHDt and GBAt/GBDt had their orthologous *UGTs* in the A and D genomes. We identified a total of 896 orthologous/paralogous gene pairs, of which 295 were predicted to be segmental duplications that form paralogous gene pairs within the GHAt/GHDt and GBAt/GBDt subgenomes. Only 71 duplication gene pairs underwent tandem duplication, while all 530 duplication gene pairs underwent WGD. Polyploidy, segmental duplication and tandem duplication were the main factors for the large-scale expansion of the *UGT* gene family.

In addition, homology analysis of *UGTs* from four cotton species showed the collinearity of the different genomes with each other. Chromosomes A-05, A-11, A-12, and A-13 contributed the most collinear genes from the A genome (*G. arboreum*) to the AD genome (*G. hirsutum* and *G. barbadense*). However, D-01, D-02, D-03, D-12, and D-13 from the D genome (*G. raimondii*) contained a higher number of collinear genes with the AD genome (*G. hirsutum* and *G. barbadense*). The A genome (*G. arboreum*) had 211 and 289 duplication gene pairs with *G. hirsutum* and *G. barbadense*, respectively. Similarly, the diploid D-genome was found to contain 311 and 310 duplicate gene pairs for each tetraploid species (*G. hirsutum* and *G. barbadense*) (Figure 7, Supplementary Table S10). Overall, we obtained 2,203 duplicated gene pairs among 752 *UGTs* in four cotton species, which laid the foundation for the polyploidy and large-scale expansion of the *UGT* gene family during evolution.

### Selection pressure analysis

To investigate the effect of Darwinian positive selection and selection pressure on the evolution of *UGTs*, we calculated the ratio of Ka and Ks for orthologous/paralogous pairs from four cotton species (Figure 8, Supplementary Table S7). We identified 1,501 orthologous/paralogous pairs from four cotton species (GH, GB, Ga, and Gr). The Ka/Ks values of 1,456 (97.00%) orthologous/paralogous pairs were all less than 1. Among them, there were 1,196 orthologous/paralogous pairs with Ka/Ks values less than 0.5 and 260 orthologous/paralogous pairs with Ka/Ks values between 0.5 and 0.99. This indicated that the *UGT* gene family underwent overall strong purifying selection during evolution, leading to similar functions. Only 45 (3.00%) orthologous/paralogous pairs had a Ka/Ks ratio greater than 1, and these orthologous/paralogous genes may have undergone relatively rapid evolution after duplication and may have undergone positive selection. The Ka/Ks values of Ga-Ga, Gr-Gr and Ga-Gr were all less than 1, indicating that the paralogous genes from diploid cotton were all selected by purifying selection. Given that most Ka/Ks values were less than 1, we speculate that the *UGT* gene family has undergone strong purifying selection pressure with limited functional differentiation after segmental duplication and WGD (Figures 8A,B, Supplementary Table S7). We further calculated the segmental duplication time periods for these genes, as shown in Supplementary Table S7. Duplications of *UGT* gene segments from *G. hirsutum* showed a duplication history between 0.42 Mya and 155.73 Mya. Similarly, segmental duplication of *UGTs* in *G. raimondii* and *G. arboreum* occurred between 20.27 Mya and 140.74 Mya and 4.05 Mya and 121.55 Mya, respectively. *G. barbadense* showed an evolutionary background from 0.41 Mya to 119.44 Mya (Supplementary Table S7).

### Gene ontology analysis of four cotton species

**FIGURE 9:**
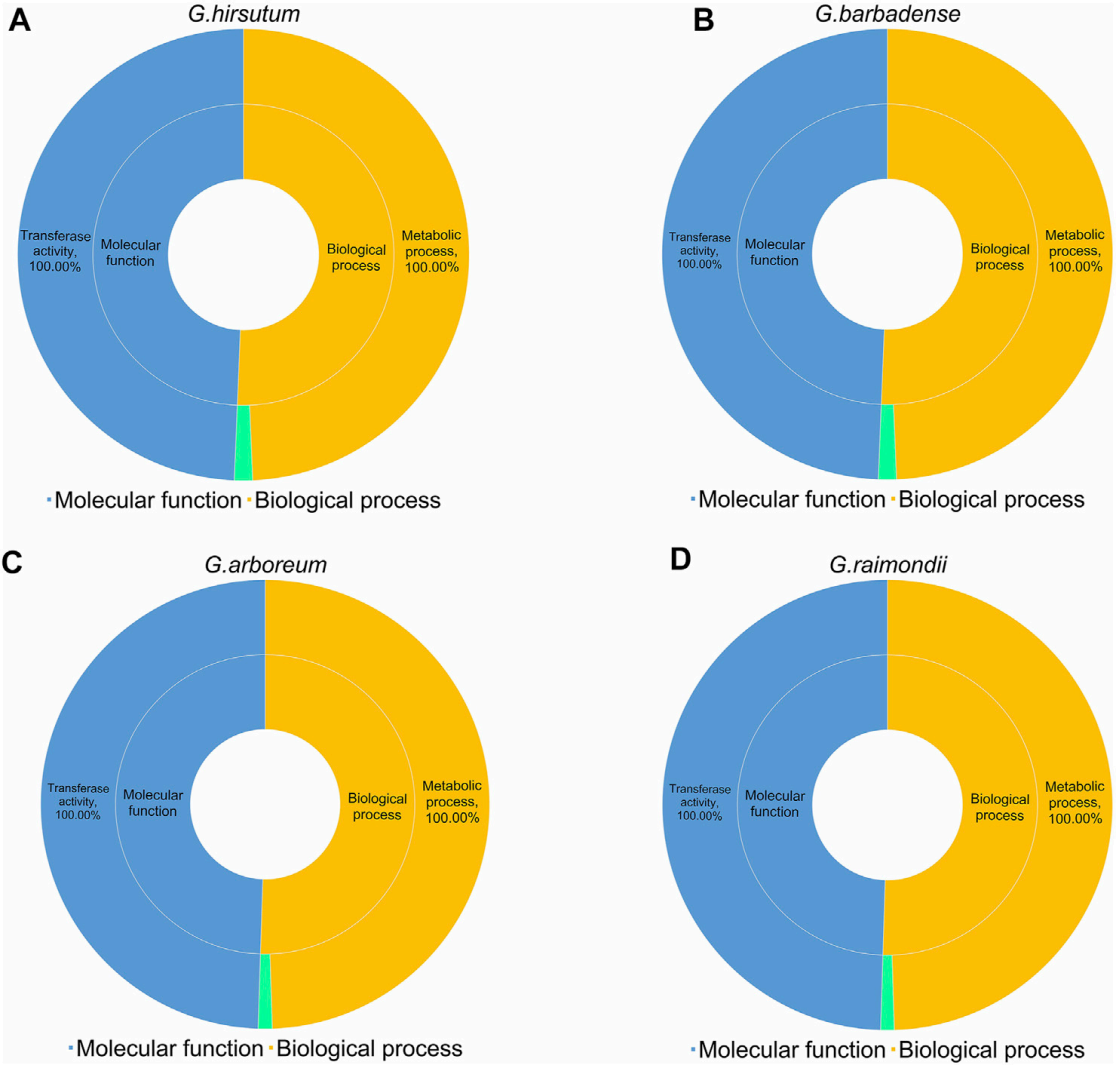
GO analysis of 752 *UGTs* from four cotton species. **(A)** *G. hirsutum.* **(B)** *G. barbadense* **(C)** *G. arboreum* **(D)** *G. raimondii*.

Gene Ontology is an internationally standardized gene function classification system that provides a dynamically updated set of controlled vocabulary to comprehensively describe the properties of genes and gene products in organisms. For GO analysis, we predicted the regulatory functions of 752 *UGTs* from four cotton species. We obtained data from CottonFGD (Zhu et al., 2017) and divided it into two categories: molecular functions and biological processes. For molecular functions, all 752 *UGTs* were involved in “transferase activity (GO:0016758)”. For biological processes, all *UGTs* were annotated to “metabolic process (GO:0008152)”, and the genes annotated to” lipid glycosylation (GO:0030259)” included six *GHUGTs* (2.65%), six *GBUGTs* (2.63%), three *GaUGTs* (2.05%), and three *GrUGTs* (1.97%). Altogether, the GO terms indicated that all 752 *UGTs* from the four cotton species were annotated to “transferase activity (GO:0016758)” and “metabolic process (GO:0008152)” (Figure 9, Supplementary Table S8).

### Analysis of diversified expression profiles and cis-acting elements

**FIGURE 10:**
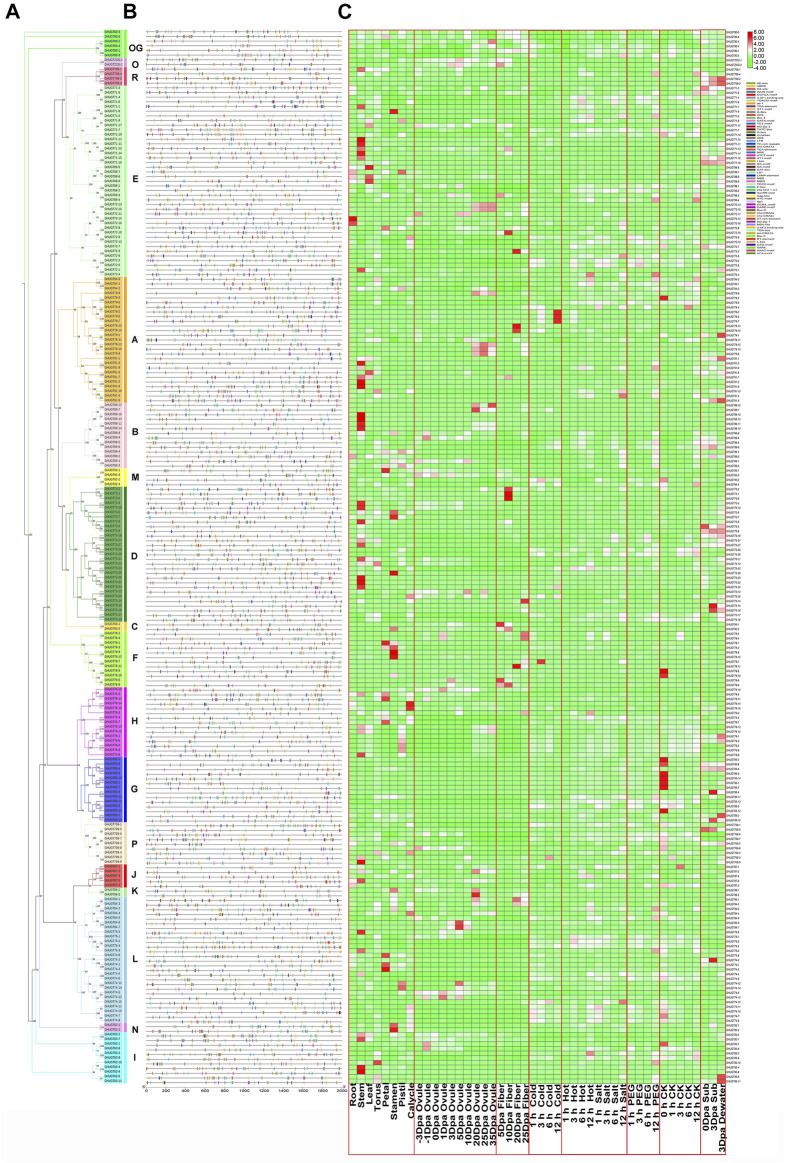
Promoters and differentially expressed gene analysis of the *GHUGT* family. **(A)** Phylogenetic tree of *GHUGTs*. **(B)** Cis-elements in the promoters of *GHUGTs*. **(C)** Differentially expressed genes of *GHUGTs* under cold, hot, salt, PEG and submergence stress and tissue-specific expression patterns. The change from green to red represents the change in gene expression level from low to high.

A cis-acting element is a sequence present in the flanking sequence of a gene that can affect gene expression and is mainly involved in the regulation of gene expression. Cis-acting elements include promoters, enhancers, regulatory sequences, and inducible elements, among others. We analysed the promoter regions of 226 *GHUGTs*, mainly including DNA sequences 2000 bp upstream of the transcription start site (TTS). In the promoter regions of the *GHUGT* gene family, we found a large number of cis-acting elements involved in various cellular physiological processes. There were differences in the cis-acting elements of *GHUGTs* from different clades. A large number of cis-acting elements related to the light response, a total of 29 cis-acting elements, were widely distributed in all clades. Box4, G-Box, GT1-motif, TCT-motif, GATA-motif, and MRE were the most abundant light-responsive elements, accounting for 90.71%, 74.34%, 73.45%, 56.19%, 45.13%, and 43.36% of the total *GHUGTs*, respectively (Figure 10 and Supplementary Table S9). A total of 9 cis-acting elements were found from the biotic/abiotic stress response category, and ARE (anaerobic induction), As-1 (low temperature responsive), WUN-motif (wound response) and LTR (low temperature responsive) were present in 83.19%, 62.83%, 56.19% and 50.88% of *GHUGTs*, respectively (Figure 10 and Supplementary Table S9). There were 11 cis-acting elements in the plant hormone response category, including ABRE (abscisic acid responsiveness), CGTCA-motif (MeJA-responsiveness), TGACG-motif (MeJA-responsiveness), TCA-element (salicylic acid responsiveness), TGA-element (auxin responsiveness) and P-box (gibberellin-responsive element), which accounted for 66.81%, 62.83%, 62.83%, 41.15%, 32.30%, and 26.55% of the total *GHUGTs*, respectively (Figure 10 and Supplementary Table S9). Similarly, 10 cis-acting elements were also found in the growth and development category. CAT-box (meristem expression) was the most abundant growth and developmental response element, accounting for 42.92% of the total number of *GHUGTs*, followed by the AT-rich element (binding site of AT-rich DNA-binding protein) (21.24%) and GCN4_motif (endosperm expression) (19.03%). Cis-acting elements related to stress, hormone response and growth and development were abundantly found in *GHUGTs*, indicating that they may play important roles in plant growth and development, plant hormone response and different stresses.

Since gene expression is closely related to cis-acting elements, we examined *GHUGTs* for abiotic stresses such as salt, drought, water, heat and cold, and tissue-specific expression patterns in roots, stems, leaves, fibres, etc. RNA-seq data were downloaded from the NCBI and analysed. RNA-Seq data from 10 tissues in *G. hirsutum* showed different expression patterns of *GHUGTs* within the same clade (Figures 10A,C). Moreover, genes from the same clade may be functionally different despite similar motifs under different abiotic stresses (Figures 9A,C). In conclusion, the expression analysis of *GHUGTs* under different abiotic stresses and in various tissues indicates that the *GHUGT* gene family is important in plant growth and development.

### Validation analysis of the expression levels of *GHUGTs*

**FIGURE 11:**
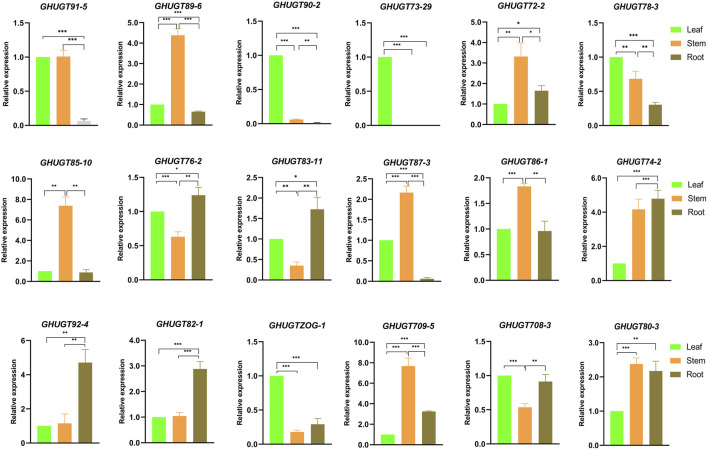
Tissue-specific expression of 18 *GHUGT* members differentially expressed genes. *: *p* < 0.05, **: *p* < 0.01 and ***: *p* < 0.001. Error bars are the standard deviation (SD) of three biological replicates in each treatment group. The statistical test is Student’s t-test.

**FIGURE 12:**
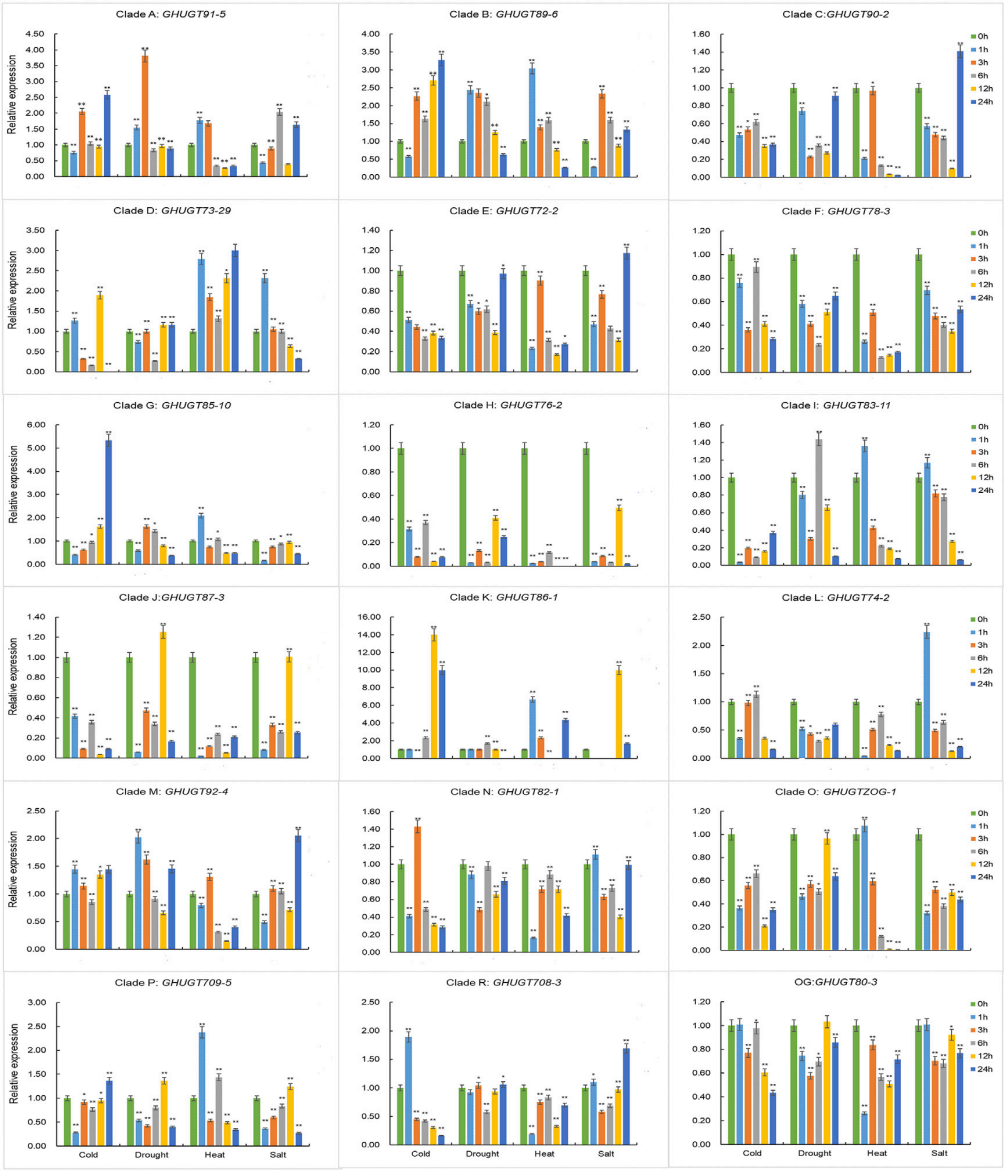
The expression levels of 18 *GHUGT* members at different times under abiotic stresses, including cold, drought, heat and salt. Error bars represent the SD between three biological replicates in each treatment group. The statistical test is Student’s t-test. *: *p* < 0.05, **: *p* < 0.01.

To further understand the tissue-specific expression profiles and responses to abiotic stresses of the *GHUGT* gene family, we analysed 18 *GHUGTs* from 18 clades. We found that 18 *GHUGTs* had different expression patterns in different tissues. For example, *GHUGT90-2*, *GHUGT73-29*, and *GHUGTZOG-1* were highly expressed in leaves and expressed at low levels in roots and stems. *GHUGT89-6*, *GHUGT85-10*, and *GHUGT87-3* were highly expressed in stems and expressed at low levels in roots and leaves. *GHUGT92-4* and *GHUGT82-1* were highly expressed in roots and expressed at low levels in leaves and stems (Figure 11). In addition, *GHUGTs* responded differently to various abiotic stresses (Figure 12). For example, *GHUGT85-10* and *GHUGT83-11* were highly expressed under cold stress but expressed at low levels under drought and heat stress; *GHUGT74-2* was only highly expressed under heat stress. The above results further verified that the GHUGT gene family may be closely related to cotton growth and development.

### Function validation of *GHUGT74-2*

**FIGURE 13:**
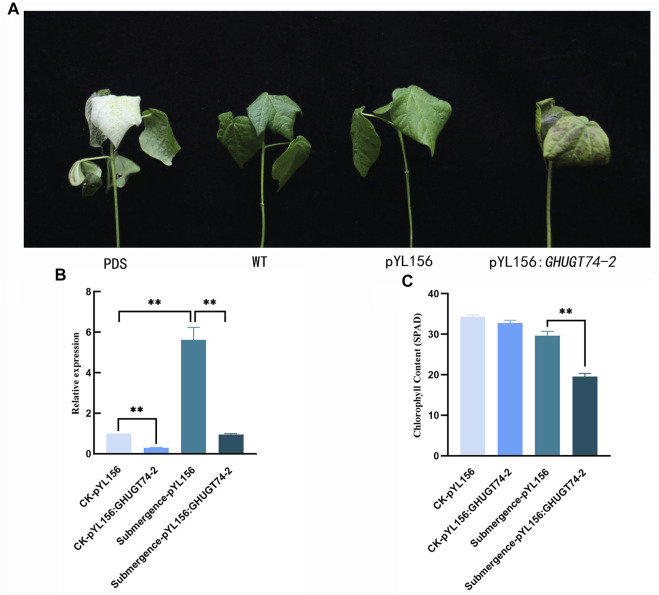
Phenotypic and biochemical indicators of cotton after *GHUGT74-2* gene silencing under submergence. **(A)** Phenotypic of cotton after *GHUGT74-2* gene silencing under submergence. **(B)** Expression level of *GHUGT74-2* in control and *GHUGT74-2*-silenced plants. **(C)** Chlorophyll content in control and *GHUGT74-2*-silenced plants. PDS: albino cotton injected with pYL 156: PDS.; WT: normal growth cotton; pYL 156: blank control cotton injected with pYL 156 vector; pYL156:*GHUGT74-2*: silenced cotton injected with pYL 156: *GHUGT74-2*. *: *p* < 0.05, **: *p* < 0.01.

Virus-induced gene silencing (VIGS) is a powerful tool for the study of gene function (Lu et al., 2003; Senthil-Kumar and Mysore, 2011). To further investigate the function of *UGT* gene in abiotic stress, gene silencing analysis of *GHUGT74-2* was performed by VIGS (Figure 13). When cotton grew to the three-leaf stage, the plants injected with pYL156:PDS appeared albino, indicating that *GHUGT74-2* was successfully silenced. Under normal growth conditions, the expression level of the silent plant (Number: pYL156:*GHUGT74-2*) was significantly lower than that of the control (Number: pYL156). When submerged for 3 days, the phenotypic changes of the plants injected with pYL 156:*GHUGT74-2* were more obvious than those of the plants injected with the pYL156 vector, and the leaves appeared more obvious wilting and browning (Figure 13A). The expression level of the silent plants (Number: pYL156:*GHUGT74-2*) was significantly lower than that of the control (Number: pYL156). At the same time, the chlorophyll content of the silent plants (Number: pYL156:*GHUGT74-2*) was significantly lower than that of the control (Number: pYL156). Studies have shown that *GHUGT74-2* helps alleviate submergence stress).

## Discussion

*UGTs* are widespread in all plants. Compared with *Arabidopsis* (Ross et al., 2001), *Zea mays* (Li et al., 2014), *Cicer arietinum* (Sharma et al., 2014), *Linum usitatissimum* (Barvkar et al., 2012), *Brassica rapa* (Yu et al., 2017), *Prunus mume* (Zhang et al., 2018), *Triticum aestivum* (He et al., 2018), *Manihot esculenta* (Wu et al., 2021), *G. hirsutum* and *G. barbadense* contain the highest number of *UGTs*. This may be because the allopolyploid *G. hirsutum* and *G. barbadense* have undergone whole-genome duplication, as well as a large number of segmental duplications and tandem duplications during evolution (Malik et al., 2022). In the present study, 226, 228, 146, and 152 *UGTs* were identified in *G. hirsutum, G. barbadense*, *G. arboreum*, and *G. raimondii*, accounting for 0.31% (226/72761), 0.30% (228/75071), 0.36% (146/40960) and 0.41% (152/37505) of the total number of genes in its genome, respectively. The large variation in sequence lengths (from 1,026 to 93336 bps) and diverse gene structures (different numbers of introns from 1 to 15) of *UGTs* from the four cotton species suggest a divergence within the *UGT* gene family. Changes in protein motifs also hint at their evolution in gene families. In recent years, with the development of cotton genome resequencing and genomics, genome assembly and annotation have become more precise, making it possible to systematically assess the evolution and structural characteristics of the *UGT* gene family in cotton.

### Structural analysis of the *UGT* gene family

Understanding the distribution of genes on chromosomes helps us to discover enriched regions of gene function. We found that most *UGTs* were distributed on both ends of the chromosome and showed a nonuniform distribution (Figure 4). *UGTs* were usually distributed on chromosomes in clusters of 2–12 genes. Genes in the same cluster showed a high degree of sequence similarity and were often grouped into the same clade. A total of 146 tandem duplication pairs were found in four cotton species (Figure 3). These gene clusters may be gene-enriched regions. Four UGT auxin-related genes were knocked out in *Arabidopsis* which are arranged in tandem in the genome (Mateo-Bonmatí et al., 2021).

Previous studies have shown that exon–intron distribution patterns in genes are related to their biological functions (Malik et al., 2020). Mainly taking the polyploid model plant allotetraploid *G. hirsutum* as the research object, the relationship between the structure and function of the *UGT* gene family was studied. Studies have shown that approximately 55.54% of *GHUGTs* have no introns, and approximately 39.94% have only one intron. *GHUGTs* of the same clade have similar structures and arrangements in terms of intron number and exon length (Figures 5A,C). There is a highly conserved sequence PSPG-box in the C-terminal region of UGT proteins, while the protein motifs in the N-terminal region vary greatly (Figure 5B). This suggests that the C-terminal region is involved in the binding of the UDP moiety of the nucleotide sugar substrate (Li et al., 2001). We found that the *GHUGT* gene family has a strong evolutionary relationship between gene structure and protein motif distribution and exhibits a conserved pattern of gene structure and protein motifs.

### Evolutionary analysis of the *UGT* gene family

Phylogenetic analysis helped us to understand the differences in *UGT* family genes and analyse their evolutionary processes. According to the criterion proposed by Alexander E. (Wilson and Tian, 2019), we divided 752 *UGTs* into 18 clades and named them the A-P, R and OG clades. The 18 clades included 14 conserved clades (A-N) that exist in *A. thaliana* (Li et al., 2001; Ross et al., 2001) and Clades O, P, R, and OG (Figure 2). We classified the glycosyltransferase genes annotated as cytokinin glycosyltransferase (*ZOG*), 7-deoxyglycine glucosyltransferase (*UGT709C*), sterol 3-β-glucosyltransferase (*UGT80A*), and cyclodextrin glycosyltransferase (*CTG*) as the O, P, OG, and R clades, respectively. O and P clades have also been found in some plants (Huang et al., 2015; Zhou et al., 2017). The OG and R clades have not been reported in cotton (Huang et al., 2015), and are newly discovered in this study. The Q clade was not found from four cotton species, which is consistent with the finding in Poales and Brassicales (Wilson and Tian, 2019). During cotton evolution, four clades (A, D, E, G, and L) expanded more than others, which is consistent with previous findings (Caputi et al., 2012). Clade E had the largest number of *UGT* members (136), accounting for 18.09% of the *UGTs*. Clade R was not found in *G. hirsutum*, which may be related to gene loss during *G. hirsutum* evolution. Gene duplication provides the raw material for gene function innovation, which facilitates not only large-scale gene amplification but also speciation and adaptation to the environment (Hittinger and Carroll, 2007; Conant and Wolfe, 2008; He et al., 2020). There are three main methods of gene amplification: WGD, segmental duplication and tandem duplication. WGD, also known as polyploidy, is one of the methods of gene amplification. Angiosperm polyploidy is closely related to evolution (He et al., 2020). Allotetraploid *G. hirsutum* is a model crop for studying polyploidy (Li et al., 2015). Our results show that allotetraploid cotton (*G. hirsutum* and *G. barbadense*) has almost double the number of *UGTs* as monodiploid cotton (*G. arboreum* and *G. raimondii*) (Figure 1). At the same time, 80.84% (1781/2,203) of duplicated gene pairs underwent WGD. This also verifies that *G. hirsutum* and *G. barbadense* were produced by interspecific hybridization between the ancestor species with the A genome and the ancestor species with the D genome (Wendel and Cronn, 2003; Paterson et al., 2012; Li et al., 2015). Interestingly, 226, 228, 146, and 152 *UGTs* were identified in *G. hirsutum, G. barbadense*, *G. arboreum*, and *G. raimondii*, respectively. The total number of *UGTs* from tetraploids was less than twice the total number of *UGTs* from diploids, suggesting that gene loss occurred during the evolution of allotetraploid cotton. Similar results have also been reported before, such as gene loss in glutaredoxin (Malik et al., 2020), bZIP transcription factor (Wang et al., 2020), choline kinase (Wang et al., 2021) and ABC protein family (Malik et al., 2022). Moreover, we obtained a total of 2,203 duplicated gene pairs in four cotton species, including 276 segmental duplications and 146 tandem duplications (Figure 6). WGD, segmental duplication and tandem duplication have all played important roles in the large-scale amplification of the cotton *UGT* gene family, which is consistent with previous reports (Wu et al., 2021).

Gene families face selection pressure after gene amplification. People often use Ka/Ks to determine which type of selection pressure plays a major role. If Ka/Ks > 1, the gene is considered to be subject to positive selection; if Ka/Ks = 1, the gene is considered to be subject to neutral evolution; if Ka/Ks < 1, the gene is considered to be subject to purifying selection. In this study, 97.00% (1,456) of orthologous/paralogous pairs had Ka/Ks ratios less than 1, indicating that *UGTs* from four cotton species had undergone a high degree of purifying selection pressure (Figures 8A,B, Supplementary Table S7). At the same time, we found 45 (3.00%) orthologous/paralogous pairs with a Ka/Ks ratio greater than 1, and these orthologous/paralogous genes experienced positive selection pressure after duplication. In general, after the *UGT* family experienced WGD, segmental duplication and tandem duplication in the evolutionary process, most genes were subjected to purification selection, and only a few genes faced positive selection pressure.

### Function analysis of the *UGT* gene family

*UGTs* play an important role in plant growth and development. *OsIAGT1* regulates rice growth and development by regulating auxin homeostasis (Liu et al., 2019). Our results show that 10 cis-acting elements are related to growth and development, mainly including CAT-box (meristem expression), AT-rich element (binding site of AT-rich DNA-binding protein) and GCN4_motif (endosperm expression), and 11 cis-acting elements are involved in the regulation of plant hormone responses. At the same time, we found that *GHUGTs* had expression specificity in different tissues. For example, *GHUGT90-2*, *GHUGT73-29* and *GHUGTZOG-1* were highly expressed in leaves, and *GHUGT89-6*, *GHUGT85-10* and *GHUGT87-3* were highly expressed in stems (Figure 11). These results may indicate that *UGTs* are involved in cotton growth and developmental processes.

The *UGT* gene family is widely involved in plant responses to biotic and abiotic stresses, such as detoxification responses (Poppenberger et al., 2003), defence responses (Meißner et al., 2008; Huang et al., 2021), drought (Li et al., 2018), low temperature (Zhao et al., 2019), etc. A total of 9 cis-acting elements were found in the biotic/abiotic stress response categories, including ARE (anaerobic induction), As-1 (low temperature responsive), WUN-motif (wound response) and LTR (low temperature responsive). (Supplementary Table S9). For example, *GHUGT74-2* was only highly expressed under high-temperature stress. *GHUGT85-10* and *GHUGT83-11* were highly expressed under cold stress and expressed at low levels under drought and heat stress. The above results further verify that the *GHUGT* gene family plays an important role in responding to biotic and abiotic stresses.

## Conclusion

**FIGURE 14:**
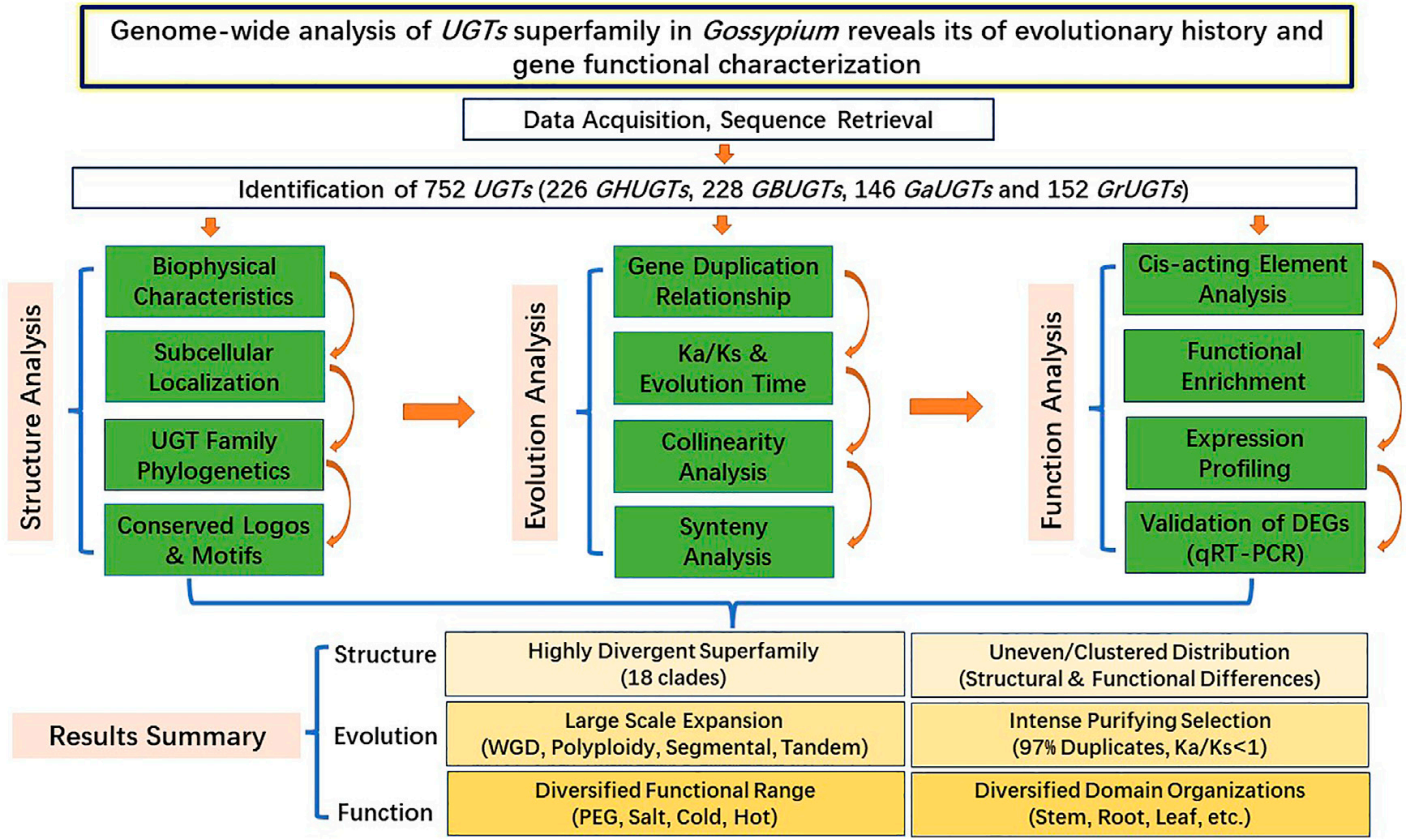
Systematic analysis diagram of *UGT* gene family structure, evolution and function.

The *UGT* gene family is one of the plant supergene families, ubiquitous in all organisms, and involved in plant growth and development and biotic and abiotic stress responses. We identified a total of 752 *UGTs* from *G. hirsutum, G. barbadense*, *G. arboreum*, and *G. raimondii* and divided them into 18 clades. Each *UGT* gene contains a conserved PSPG motif. The chromosomal distribution, biochemical properties, conserved motifs, and exon/intron location features of the *UGT* gene family provide a useful material (structural) basis for understanding the function of the *UGT* gene family. Orthologue/paralogue comparison, selection pressure analysis, and phylogenetic analysis help to understand the evolutionary processes of the *UGT* gene family. Analysis of GO enrichment, RNA-seq data, cis-acting elements, qRT–PCR, and subcellular localization can help understand the function of the *UGT* gene family. Through the joint analysis of the structure–function evolution of the gene family, this study is helpful for understanding the evolutionary processes of the cotton *UGT* superfamily and the evolutionary mechanisms of polyploidy formation and for providing a new way to systematically and globally understand the structure–function relationship of multigene families (Figure 14).

## Materials and methods

### Databases

We downloaded the latest genome and protein files of *G. arboreum* (CRI, Ver. 1.0), *G. raimondii* (JGI, Ver. 2.0), *G. hirsutum* (ZJU, Ver. 2.1) and *G. barbadense* (ZJU, Ver. 1.1) from the Cotton Functional Genomics Database (CottonFGD) (https://cottonfgd.org/) (Zhu et al., 2017). The representative reference sequences file of *Arabidopsis thaliana* was retrieved from TAIR 10 (http://www.arabidopsis.org/) (Wilson and Tian, 2019).

### Identification of *UGTs*

The known conserved domain of UGT proteins is PF00201.20 (Huang et al., 2015). The hidden Markov model (HMM) (Ver. 35.0) profile of PF00201.20 was downloaded from the online website Pfam (https://pfam.xfam.org/). The *UGT* gene containing PF00201.20 was obtained as a candidate gene for a *UGT* gene family member using native software HMMER 3.0. We retained redundant genes with e-values less than 1E-05. We further assessed our genes by NCBI Batch CD-Search (https:// www.ncbi.nlm.nih.gov/Structure/bwrpsb/bwrpsb.cgi) (Lu et al., 2020) and manually deleted genes with incomplete C and N ends. Based on the conserved PF00201 domain of the *UGT* gene family, the protein sequences of UGTs from four cotton species were screened and downloaded from CottonFGD (Zhu et al., 2017). According to the location of *UGTs* on the chromosome, we renamed *UGTs* of *G. hirsutum* (GH), *G. barbadense* (GB), *G. arboreum* (Ga), *G. raimondii* (Gr), respectively (Supplementary Table S1). We further retrieved various biophysical and chemical properties of *UGTs* from four cotton species by using CottonFGD, including transcript lengths, exon/intron lengths, molecular weights (MWs), protein lengths, grand average hydropathy and charge (Supplementary Table S2) (Zhu et al., 2017).

### Phylogenetic and sequence alignments

The UGT amino acid sequences of the four plant species are given in Supplementary Table S3. We used ClustalW (Ver. 2.0) with the default setting for amino acid sequence complete alignments (Larkin et al., 2007) and then constructed the phylogenetic tree in Toolbox for Biologists software (TBtools, Ver. 1.098693) using the maximum likelihood (ML) method with 5,000 bootstrap number (Chen et al., 2020).

### Chromosomal locations of *UGTs*

Gene annotation files (GFF3 format) of four cotton species were obtained from CottonFGD (Zhu et al., 2017). Toolbox for Biologists software (TBtools, Ver. 1.098693) was used to visualize the physical locations of *UGTs* on chromosomes from four cotton genomes (Chen et al., 2020).

### Analysis of the conserved protein motifs and gene structure

We used the online webtool MEME (Ver. 5.4.1) (https://meme-suite.org/meme/tools/meme) to predict the conserved protein motifs of UGT proteins (Bailey et al., 2009). A GHUGT phylogenetic tree, conserved motifs and gene structures were drawn with TBTools (Ver. 1.098693) by using a NWK file for the phylogenetic tree, the gff3 file of *G. hirsutum* and MAST file obtained from the MEME (Ver. 5.4.1) website (Bailey et al., 2009; Chen et al., 2020).

### Collinearity analysis of *UGTs*

Synteny relations between duplicated gene pairs from *G. barbadense, G. hirsutum*, *G. arboreum* and *G. raimondii* were analysed by MCScanX software (Wang et al., 2012). The graphical results were displayed using TBtools software (Chen et al., 2020).

### Analysis of selection pressure

Duplicated gene pairs of four cotton species were obtained by using alignment in MEGA 7.0. We calculated the nonsynonymous (Ka) and synonymous (Ks) substitution rates of *UGT* duplicated genes to investigate the selection pressure using TBtools software (Chen et al., 2020). We calculated the evolutionary time of duplicate pairs using the following formula: T = Ks/2λ × 10^–6^ (Mya) and λ = 1.5 × 10^–8^ (Malik et al., 2022).

### Cis-acting element analysis and gene expression profiling of *GHUGTs*

DNA sequences in upstream 2000 bp regions of *GHUGTs* were downloaded from CottonFGD (https://cottonfgd.org) (Zhu et al., 2017) as promoters. We used the oneline site PlantCARE (http://bioinformatics.psb.ugent.be/webtools/plantcare/html/) to predict cis-acting elements in *GHUGT* promoter regions. We further analysed cis-acting elements related to light response, plant growth and development, phytohormones and abiotic stresses.

RNA-Seq data (accession: PRJNA780360, PRJNA248163) were downloaded from the NCBI database (https://www.ncbi.nlm.nih.gov/) to examine the expression profiling of *GHUGTs* in different tissues, including stem, root, leaf, torus, petal, stamen, pistil, calycle, ovule and fibre development, and under abiotic stress, including PEG, salt, cold, submergence and heat stress, with various time laps (Hu et al., 2019). We obtained heatmaps with FPKM values for *UGT* relative expression analysis by TBtools software (Chen et al., 2020).

### qRT–PCR analysis of *GHUGTs*

Tissue-specific expression profiles of different *GHUGTs* in roots, stems, and leaves and responses of different *GHUGTs* to abiotic stresses, such as salt (250 mM NaCl), drought (15% PEG), cold (-4 C°), and heat (42 C°) at different time intervals (0 h, 1 h, 3 h, 6 h, 12 h, and 24 h), were analysed by qRT–PCR. ZNL2067 seeds were sown in a medium (sand and vermiculite mixed at 1:1.5) and cultivated in a light incubator (25°C, 16 h/8 h day/night) until the three-leaf stage. Abiotic stress treatment and tissue sampling of roots, stems, and leaves were performed at the three-leaf stage, and each experiment used three independent biological replicates. We used the EASYspin Plus Plant RNA Kit (Aidlab Co., LTD., Beijing, China) to extract total RNA from cotton samples and then synthesized cDNA following the instruction manual of TransStart Top Green qPCR SuperMix (TransGene Biotech Co., LTD., Beijing, China). RT–qPCR primers for 18 *GHUGTs* were designed on the online tool GenScript (https://www. genscript. com/tools/real-time-pcr-taqman-primer-design-tool). All primer sequences of 18 *UGTs* are given in Supplementary Table S4. RT–qPCR experiments were performed on the Bio–Rad 7,500 rapid real-time PCR platform. The relative expression of *GHUGTs* was calculated using the 2^−ΔΔCt^ method. VIGS Technology refers to Zhang Yuexin et al. (Zhang et al., 2022).

## Data Availability

The original contributions presented in the study are included in the article/Supplementary Material, further inquiries can be directed to the corresponding author.

## Abbreviations

GO, Gene Ontology; UGT, UDP-glycosyltransferase; PSPG, Plant secondary product glycosyltransferase; GT, Glycosyltransferase; UFGT, Flavonoid glucosyltransferase; *Gossypium hirsutum*, *G. hirsutum*; *Gossypium arboreum*, *G. arboreum*; *Gossypium raimondii*, *G. raimondii*; *Gossypium barbadense*, *G. barbadense*
