# Supplementary figures and images for: Genome-wide identification, evolution and function analysis of UGTs superfamily in cotton
Source: Front Mol Biosci. 2022 Sep 13;9:965403. doi: 10.3389/fmolb.2022.965403 (PMC9513525; doi:10.3389/fmolb.2022.965403)

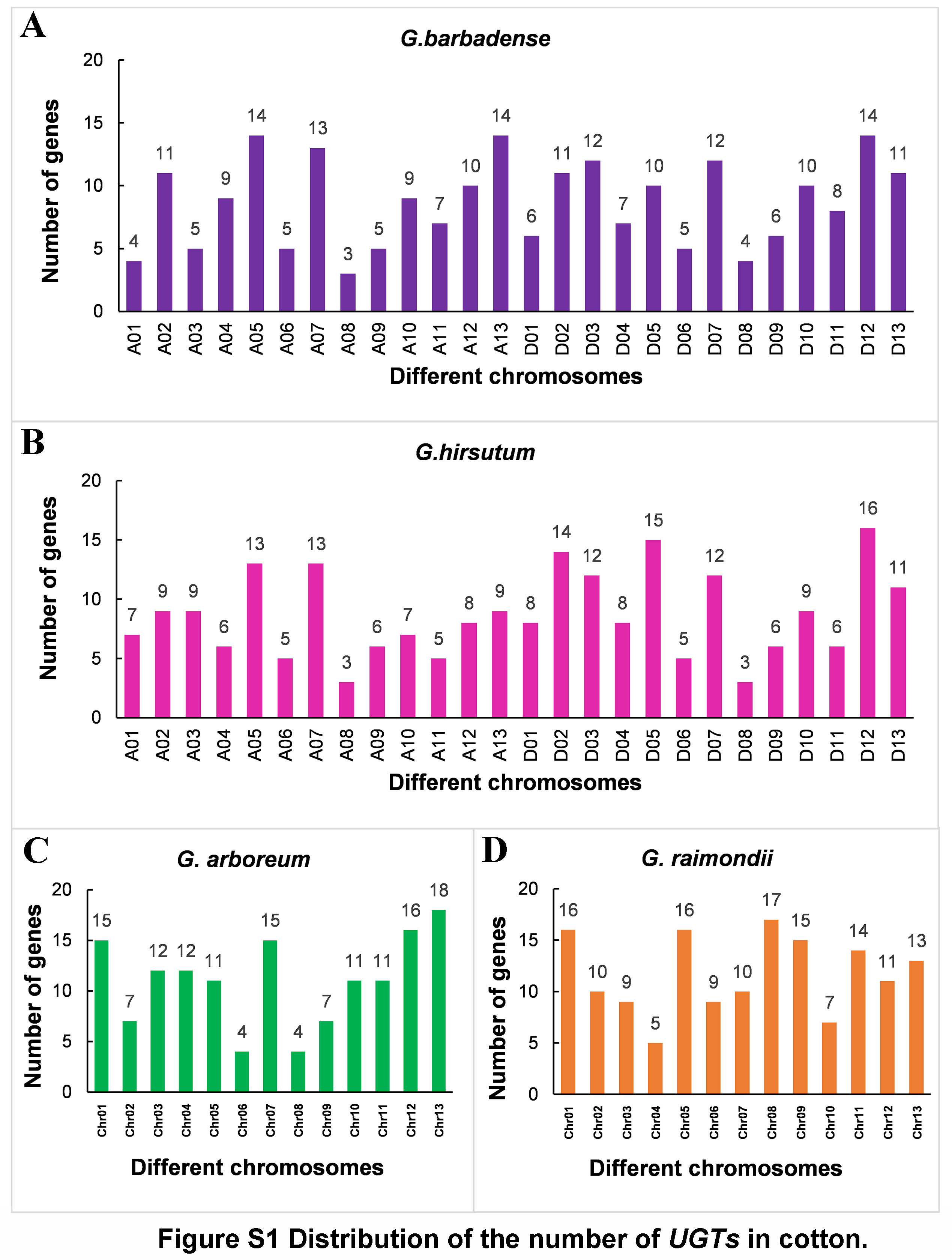

Supplement: Supplementary file 11 [file Image1.jpg]
